# The Influence of Nutrition on Adiponectin—A Narrative Review

**DOI:** 10.3390/nu13051394

**Published:** 2021-04-21

**Authors:** Justyna Janiszewska, Joanna Ostrowska, Dorota Szostak-Węgierek

**Affiliations:** Department of Clinical Dietetics, Faculty of Health Sciences, Medical University of Warsaw, E Ciołka Str. 27, 01-445 Warsaw, Poland; jjaniszewska@wum.edu.pl (J.J.); dorota.szostak-wegierek@wum.edu.pl (D.S.-W.)

**Keywords:** adiponectin, high-molecular-weight adiponectin, diet, Mediterranean diet, Western diet, eating pattern

## Abstract

The adipose tissue is an active endocrine organ which synthesizes and secretes a variety of adipokines, including adiponectin with its anti-inflammatory properties. Its expression is influenced by numerous factors such as age, sex, body weight and adipose tissue content. However, dietary factors, i.e., diet structure and the percentage of individual nutrients and products, are very important modulators. Beneficial dietary habits are the Mediterranean diet, DASH diet, diet based on plant products and diet with reduced energy value. Moreover, the share of individual products and nutrients which increase the concentration of adiponectin is worth noting. This group may include monounsaturated fatty acids, polyunsaturated omega-3 fatty acids, dietary fiber, polyphenols, alcohol and milk products. Conversely, dietary ingredients which have a negative effect on the concentration of adiponectin are typical components of the Western diet: saturated fatty acids, trans fatty acids, monosaccharides and disaccharides, and red meat. Furthermore, a diet characterized by a high glycemic index such as a high-carbohydrate low-fat diet also seems to be unfavorable. Due to the fact that available knowledge should be systematized, this study aimed to summarize the most recent research on the influence of dietary factors on the concentration of adiponectin.

## 1. Introduction

The adipose tissue, being the reservoir of energy, is also an active endocrine organ which synthesizes and secretes a variety of adipokines influencing the regulation of human metabolism. Adiponectin is one of the most important adipokines. It is a bioactive peptide composed of 244 amino acids constituting approximately 0.01% of plasma proteins. Plasma adipokine occurs in three types of complexes: (i) low-, (ii) medium-, and (iii) high-molecular-weight. High-molecular-weight (HMW) adiponectin is considered to be the most common and active form of adiponectin [1]. Furthermore, the activity of this adipokine also depends on the appropriate ratio between low- and high-molecular-weight adiponectin [2]. Currently, two isoforms of the adiponectin receptor are known: AdipoR1 and AdipoR2, which are located mainly in the skeletal muscles and the liver [1].

Adiponectin presents antineoplastic, cardioprotective and anti-inflammatory properties [3]. Additionally, it sensitizes tissues to insulin activity which contributes to its hypoglycemic properties [1]. Its hypolipidemic properties involve increasing the oxidation of fatty acids, reducing the storage of triglycerides in the skeletal muscles and increasing high density lipoprotein in the plasma via the activation of PPARα (Peroxisome Proliferator-Activated Receptor α). Its hypoglycemic effect is mostly due to the activation of PPARα, AMPK (AMP-Activated Protein Kinase), glucose transporters in the cell membrane such as GLUT4 (Glucose Transporter Type 4) and the reduction of gluconeogenesis in the liver [4,5].

Adiponectin expression is influenced by numerous factors including age, physical activity and ethnicity. Factors related to sex are also important determinants of its concentration. Women are characterized by a higher concentration of adiponectin compared to men, which is mainly due to the presence of different sex hormones [6]. Genetic factors also seem to be particularly important, because the concentration of adiponectin can be inherited by up to 55% [7]. Moreover, body weight and BMI (Body Mass Index) are also strongly correlated with adiponectin concentrations. Cruz-Mejía et al. [8] observed that adiponectin concentrations were markedly lower in obese individuals compared to participants with normal body weight (16.03 ± 2.53 μg/mL vs. 28.18 ± 1.97 μg/mL; *p* = 0.01). In addition, adiponectin concentrations were negatively correlated with the degree of obesity in obese patients (*r* = −0.477; *p* = 0.001). Interesting results were obtained in the POUNDS Lost Trial [9] which revealed statistically significant correlations between adiponectin concentrations, body composition and adipose tissue distribution. Increased adiponectin concentrations were significantly correlated with the reduction in the total adipose tissue (*β* = −0.68; *p* = 0.005), adipose tissue located within the trunk (*β* = −0.57; *p* = 0.005), subcutaneous adipose tissue (*β* = −0.42; *p* = 0.002) and visceral adipose tissue (*β* = −0.22; *p* = 0.02). Similar results were obtained by Gariballa et al. [10]. They demonstrated that the increased amount of visceral adipose tissue was related to the reduction in total adiponectin concentration. Furthermore, Meshkini et al. [11] noted that the adipose tissue content within the trunk was negatively correlated with adiponectin values (*r* = −0.44; *p* < 0.001). A high amount of adipose tissue in the area was also a strong prognostic factor of adiponectin concentration (*β* = −0.487; *p* < 0.001).

Apart from the above mentioned factors, adiponectin concentration is also influenced by dietary patterns and the share of individual products and nutrients in the diet. An appropriate dietary structure seems to be one of the most important factors increasing adiponectin concentrations. Beneficial dietary habits are the Mediterranean diet (MD), DASH diet (Dietary Approach to Stop Hypertension), diet based on plant products and diet with a reduced energy value. The products and nutrients which increase adiponectin concentrations include monounsaturated fatty acids, polyunsaturated omega-3 fatty acids, dietary fiber, polyphenols, alcohol and milk products. Conversely, dietary ingredients which have a negative effect on the concentration of adiponectin are saturated fatty acids, trans fatty acids, monosaccharides and disaccharides, and red meat, which are typical components of the Western diet. Furthermore, a diet characterized by a high glycemic index and a high-carbohydrate low-fat diet also seem to be unfavorable [1,2,12].

Due to the fact that available knowledge should be systematized, this study aimed to summarize the most recent research on the influence of dietary factors on the concentration of adiponectin.

## 2. Diet-Related Factors with a Positive Influence on Adiponectin Concentrations

### 2.1. Dietary Structure

As regards factors which influence adiponectin expression, a key role is attributed to those which are associated with dietary habits and adhering to a healthy dietary pattern. The prospective Nurses’ Health Study [13] including 1922 women revealed that total adiponectin concentrations were 24% higher and HMW adiponectin concentrations were 32% higher in women from the highest quartile of adherence to the Alternate Healthy Eating Index (AHEI) compared to the women from the lowest quartile. Similar results were obtained by Volp et al. [14] who demonstrated a direct significant correlation between Healthy Eating Index and adiponectin concentrations.

#### 2.1.1. The Mediterranean Diet

The Mediterranean diet is one of the healthiest dietary patterns. Numerous observational and interventional studies have shown its correlation with increased adiponectin concentrations [5,15,16,17,18,19]. A traditional Mediterranean diet is characterized by a plentiful supply of vegetables, fruit, olive oil, fatty saltwater fish, whole-grain cereal products, moderate alcohol consumption and low red meat consumption [16]. It is suggested that the favorable activity of the MD on adiponectin expression may be due to the structure of the diet and the synergistic activity of its bioactive components, such as omega-3 fatty acids, fiber, vitamins and polyphenols which have a positive influence on adiponectin concentrations [20].

The ATTICA epidemiological study [4] demonstrated that adiponectin concentrations were 41% higher in persons from the highest tertile of adherence to the MD diet compared to those from the lowest tertile. Moreover, the score obtained for the diet was significantly correlated with adiponectin concentrations, both in women and in men. The described correlation was confirmed with a systematic review and a meta-analysis of 20 interventional studies in which adherence to the MD was associated with a significantly higher increase in adiponectin concentration compared to the control diet [21]. Comparable outcomes were also obtained by Sureda et al. [16] in a group of 598 inhabitants of the Balearic Islands. Significantly higher adiponectin concentrations were observed in adult men who adhered to the MD compared to non-adherent men. However, such a correlation was not observed in women and adolescents of both sexes. 

Mantzoros et al. [17] conducted a study in a group of women with diabetes. They demonstrated that serum adiponectin concentrations significantly improved as a result of the MD in these women. Interesting results were also obtained by Spadafranca et al. [20] who studied the changes in serum adiponectin concentrations in 99 pregnant women in terms of the degree of adherence of the dietary pattern to the MD. Women from the highest tertile of adhering to MD were characterized by a significantly lower decrease in the percentage of adiponectin concentrations during pregnancy compared to women from the lowest tertile. 

The Mediterranean diet is associated with numerous benefits in both sexes, including those related to adiponectin concentrations, despite substantial differences between sex and the response to the MD [22]. This was corroborated by a prospective cohort study conducted by Kouvari et al. [18] in a group of 1514 men and 1528 women. Serum adiponectin was markedly improved in both sexes after the introduction of the MD. Additionally, participants with a higher degree of adherence to the MD were at a lower risk of developing liver steatosis, which was strongly correlated with adiponectin levels. The MÉDITA randomized trial [15] was conducted in 215 T2DM (Type 2 Diabetes Mellitus) patients whose adiponectin concentrations increased by 43% after a year of following the MD. A similar correlation was observed for HMW adiponectin. Furthermore, a study by Luisi et al. [19] confirmed that the beneficial effect of the MD, additionally enhanced with 40 g of extra virgin olive oil daily, on adiponectin concentrations was independent of body weight, because a significant increase in adiponectin concentrations was observed both in participants with normal body weight and with excessive body weight. The authors suggested that olive oil contributed to the strong anti-inflammatory effect of DM.

#### 2.1.2. The DASH Diet

The DASH diet is another healthy dietary pattern which, if adhered to, is associated with less severe systemic inflammation [23,24]. The DASH diet is based on vegetables, fruit, nuts, seeds of pulses, whole-grain cereal products and low-fat milk products. It is also characterized by a low content of red processed meat, sweetened beverages and products with high sodium content [25]. The mechanism through which the diet may be associated with an increased adiponectin concentration may, similarly to the Mediterranean diet, result from the presence of bioactive components with strong anti-inflammatory properties, such as polyphenols and omega-3 fatty acids. Nilsson et al. [25] conducted a study in 122 elderly women. Serum adiponectin concentrations were 20% higher in women from the highest tertile of the adherence to the DASH diet compared to women from the lowest tertile. AlEssa et al. [26] demonstrated an increasing tendency of adiponectin concentrations together with increased adherence to the DASH diet. However, the correlations were on the border of statistical significance. A beneficial effect of the diet on adiponectin concentrations may also be related to low sodium supply. According to Prates et al. [27] the dietary content of sodium was negatively correlated with adiponectin concentrations (*r* = −0.19, *p* = 0.03). Despite the paucity of studies linking this dietary pattern with adiponectin concentrations, it may be speculated that the diet may have a positive influence on the concentrations of this adipokine.

#### 2.1.3. Plant-Based Diet

A plant-based diet is another dietary pattern which may exert a beneficial effect on serum adiponectin concentrations. It may be presumed that plant-based diets have a positive effect on adiponectin concentrations, but this is not as explicit as in the case of the MD. Adiponectin may also be influenced by bioactive components. Low animal protein and total protein content seem to be beneficial in terms of adipocyte function [28,29]. Kahleova et al. [30] noted that the concentrations of total adiponectin and HMW adiponectin increased by 19% and 15%, respectively, compared to baseline in the study group which followed a vegetarian diet for 24 weeks. Furthermore, a case-control study conducted in healthy non-obese adults revealed adiponectin concentrations to be significantly higher in women following a vegetarian diet than in those following a traditional diet. However, such a correlation was not observed in men [31]. The influence of reproductive hormones on the regulation of adiponectin concentrations may be a possible mechanism explaining why the described correlation was observed only in women [11]. A cross-sectional study conducted in a group of women showed that adiponectin concentrations were strongly correlated with FSH and SHGB concentrations [32]. Besides, sex-related differences in the expression of adiponectin may depend on differences in the distribution of the adipose tissue in men and women [11]. 

However, according to some authors, plant-based diets did not influence adiponectin concentrations [29,33,34] or might be associated with its lower expression [35]. Conversely, the authors explained the described correlation by the fact that the study was conducted in India in a group of 464 women (261 vegetarians and 203 non-vegetarians), where traditional vegetarian diet was characterized by a very high consumption of carbohydrates and a low consumption of fats (including omega-3 fatty acids). It was stated that various proportions between those macronutrients contributed to the difference between the studied groups of women. In addition, systematic reviews and meta-analyses of cross-sectional [36], interventional [37] and observational studies [38] demonstrated that vegetarian and vegan diets were associated with total lower inflammation compared to the traditional diet. However, no statistically significant relationship was found between this dietary pattern and adiponectin concentrations. Ambroszkiewicz et al. [29] revealed that children following a vegetarian diet were characterized by a significantly higher ratio of anti-inflammatory adiponectin and proinflammatory leptin compared to children consuming products of animal origin, which also indicated the anti-inflammatory properties of the diet.

#### 2.1.4. Low-Energy Diet

It was also demonstrated that low-energy diets had a beneficial effect on adiponectin concentrations. It seems particularly beneficial to follow the negative energy balance pattern for a prolonged time, which resulted in effective reduction in body weight [2]. Monda et al. [39] conducted a study in 20 obese men and women. They observed that an eight-week balanced low-calorie ketogenic diet contributed to a significant increase in adiponectin concentrations both in women and in men. Furthermore, an increase was observed for all types of adiponectin of various molecular weights. 

A randomized case-control study including 107 obese adults also showed that the reduction in calorie content by 500–700 kcal contributed to a significant increase in adiponectin concentrations. A similar correlation was observed in a group of individuals using diet combined with physical activity [40]. Similar results were also obtained by Christiansen et al. [41] and Abbenhardt et al. [42]. The observed correlations were confirmed by a systematic review and meta-analysis of 13 interventional studies which demonstrated that a low-calorie diet might considerably increase adiponectin concentrations. Particularly beneficial effects were observed if the diet was followed for at least 16 weeks. The authors suggested that the beneficial effect of the reduced-calorie diet on adiponectin concentrations depended predominantly on its duration and the degree of body weight reduction [43]. 

Song et al. [44] demonstrated that adiponectin concentrations significantly increased with the degree of body weight loss. Moreover, the activation of the PPARαreceptor and the reduction of inflammation resulting from the low-calorie diet seem to underlie this correlation. Alternatively, it is believed that body weight reduction may strengthen the expression of adiponectin receptors in the liver and skeletal muscles [43].

### 2.2. Nutrients and Products Included in the Diet

Apart from healthy dietary patterns the regulation of serum adiponectin concentrations also depends on individual nutrients, i.e., monounsaturated fatty acids, polyunsaturated omega-3 fatty acids, fiber, polyphenols and products included in the diet, i.e., dairy or alcohol. Seemingly, both physical activity and the use of low-energy diets influence adiponectin concentrations, mostly via the influence on body weight. However, the influence on adiponectin concentrations seems to be direct in case of some the nutrients such as monounsaturated fatty acids or polyunsaturated omega-3 fatty acids [11].

#### 2.2.1. Monounsaturated Fatty Acids and Polyunsaturated Omega-3 Fatty Acids

Omega-3 acids, including eicosapentaenoic acid (EPA) and docosahexaenoic acid (DHA), seem to be of particular importance in the context of adiponectin concentrations. Their main dietary sources are fatty saltwater fish and seafood [45]. The mechanism through which omega-3 acids induce adiponectin expression is mostly associated with PPARγ activation, the increased expression of adiponectin genes and the inhibition of the receptors of calcium ion channels [46,47]. Moreover, omega-3 acids reduce the concentrations of TNFα (Tumor Necrosis Factor α) and IL-6 (Interleukin 6), which inhibit the activity of the gene of this adipokine [48].

A randomized case-control study by Mazaherioun et al. [49] revealed that adiponectin concentrations significantly increased compared to baseline values in a group of individuals supplementing omega-3 fatty acids at a dose of 2.7 g/day. Furthermore, the study showed that PUFA (Polyunsaturated Fatty Acids) supplementation increased the expression of AdipoR1 and AdipoR2, adiponectin receptor genes, in persons with T2DM. Similar results were obtained by Barbosa et al. [48] who demonstrated that omega-3 supplementation at a dose of 3 g/day was also associated with significantly increased adiponectin concentrations. A study by Khorrami et al. [50] conducted in patients with atrial fibrillation showed that adiponectin concentrations significantly increased as a result of eight weeks of daily supplementation with 2 g of fish oil compared to the placebo arm. Similarly, Balfegó et al. [51] conducted a study in individuals who enhanced their diet with 100 g of sardines for 6 months. Adiponectin concentrations significantly increased from 2.1 ± 0.3 μg/mL at baseline to 3.0 ± 0.3 μg/mL after six months (*p* = 0.04). Interesting results were obtained by Song et al. [52] who demonstrated that adiponectin concentrations increased in each of the three groups over the period of 12 weeks. The groups differed in terms of the dose of EPA and DHA (gr. 1–3.1 g/d; gr. 2–6.2 g/d; gr. 3–12.4 g/d). However, the highest increase in adiponectin concentrations was observed in the group in which the dose of PUFA was the highest. This suggests that the amount of consumed omega-3 acids might also be of importance as regards the influence on adiponectin concentrations.

Similar correlations were also observed in women with PCOS [53,54]. Furthermore, the beneficial effect of omega-3 acids on adiponectin was observed in women with insulin resistance and with normal sensitivity to insulin [53]. The described correlations between omega-3 acids and adiponectin concentrations were confirmed by systematic reviews and meta-analyses of randomized case-control studies [45,46].

Linseed is another source of omega-3 acids. Linseed exerts a positive effect on adiponectin expression because it contains alpha-linolenic acid (ALA), which may also act as a ligand for the PPARγ receptor. Haidari et al. [55] conducted a randomized study in a group of women with PCOS. They demonstrated that linseed might significantly increase adiponectin concentrations. A significant increase in adiponectin concentrations was observed in comparison with baseline values in a study group which enhancing their diet with 30 g of ground linseed for 12 weeks. Gomes et al. [56] also demonstrated that the supplementation with 3 g of ALA significantly increased serum adiponectin concentrations after 60 days. However, a systematic review and a meta-analysis of seven randomized case-control studies revealed no statistically significant correlation between adiponectin concentrations and linseed consumption [57]. 

Notably, other studies revealed an important role of polyunsaturated omega-6 fatty acids and monounsaturated fatty acids on the regulation of adiponectin concentrations. A study by Kalgaonkar et al. [58] conducted in a group of women with PCOS showed that both walnuts and almonds significantly increased adiponectin concentrations. The results suggested that both linoleic acid found in walnuts and oleic acid found in almonds had a positive effect on adipokine concentrations. The described correlation was confirmed by a systematic review and meta-analysis of three randomized case-control studies which showed that walnuts were a dietary component with the potential of increasing adiponectin concentrations [59]. It is worth noting the results of a study by Kabiri et al. [60] conducted in obese women, which revealed that a diet rich in olive oil had a tendency to increase adiponectin concentrations (correlation on the border of statistical significance (*p* = 0.06)).

Furthermore, suitable ratios of dietary omega-3 and omega-6 acids [61] and polyunsaturated and saturated fatty acids [13] seem to be important factors. Fargnoli et al. [13] demonstrated significantly higher total adiponectin and HMW concentrations in women with the lowest ratio of polyunsaturated to saturated fatty acids compared to women with the highest ratio.

#### 2.2.2. Dietary Fiber

Dietary fiber is another component of food which has a positive effect on adiponectin concentrations. A review of 52 studies conducted by Silva et al. [2] showed that the presence of fiber in the diet contributed to an increase in adiponectin concentrations, even up to 60–115%. The prospective Nurses’ Health Study [13] demonstrated that women from the highest quartile of cereal fiber consumption had significantly higher total adiponectin and HMW adiponectin concentrations compared to women from the lowest quartile. A cross-sectional observational study by Pereira et al. [62] showed that a higher consumption of fiber included in vegetables and fruit was associated with higher adiponectin concentrations. The concentrations of adiponectin were 4.7 μg/mL (*p* = 0.03) higher in individuals from the highest quartile of cereal fiber consumption compared to participants from the lowest quartile. 

The cross-sectional Health Professionals Follow-up Study [63], which included 780 men with T2DM, revealed that adiponectin concentrations were significantly higher in men from the highest quartile of cereal fiber consumption compared to men from the lowest quartile. Notably, the correlations between total fiber and vegetable fiber consumption and adiponectin concentrations were not statistically significant. Similar results were obtained by AlEssa et al. [64], who demonstrated that the consumption of both total fiber and fiber from cereals, vegetables and fruit was positively associated with adiponectin concentrations. Similar results were also obtained by Mantzoros et al. [17]. They observed that the consumption of whole-grain cereal products which were the source of dietary fiber was associated with significantly higher adiponectin concentration. Such an explicit correlation was also observed in the case of the consumption of fruit and nuts which also constitute an important source of dietary fiber. The seeds of pulses, including lentils, chickpeas, beans, broad beans and soy, are also excellent sources of both soluble and insoluble dietary fiber [65]. A study by Mirmiran et al. [65] conducted in a group of T2DM patients demonstrated that a diet in which two servings of red meat were replaced with pulse seeds effectively increased adiponectin concentrations.

#### 2.2.3. Polyphenols

Adiponectin concentrations and the expression of its receptors seem to be influenced by polyphenols which are secondary plant metabolites characterized by strong anti-inflammatory properties [66]. Coffee and green tea are rich sources of polyphenols. Caffeine and the catechin it includes have a beneficial effect on adiponectin mainly through the stimulation of the PPARγ receptor expression [67]. Some studies have revealed a correlation between the consumption of coffee and adiponectin concentrations [68,69,70]. A cross-sectional study conducted in Japan included 665 men. It revealed that higher coffee consumption was associated with higher adiponectin concentrations [71]. Similar results were also observed in relation to green tea [72,73,74]. Moreover, a study by Fragopoulou et al. [5] demonstrated that adiponectin concentrations were positively correlated with green tea consumption. However, a systematic review and a meta-analysis of 14 randomized case-control studies revealed no statistically significant correlation between serum adiponectin concentrations and green tea consumption. Nevertheless, the authors suggested that this might be due to the high heterogeneity of the analyzed studies (I2 D 91.7%; *p* < 0.0001) [75]. 

Curcumin is another polyphenol which has a beneficial effect on the expression of adiponectin [76,77]. Mirhafez et al. [78] conducted a study in a group of patients with nonalcoholic fatty liver disease and found that supplementation with curcumin for eight weeks contributed to a significant increase in adiponectin concentrations. Comparable results were also obtained by Adibian et al. [79] whose 10-week study included 44 patients with T2DM. The described correlations were confirmed by systematic reviews and meta-analyses of randomized case-control studies which showed a significant increase in adiponectin concentrations resulting from curcumin supplementation [80,81]. Furthermore, the highest increase was observed in case of interventions of at least 10-week duration [81].

It may be presumed that other polyphenols also increase the expression of adiponectin, but the data are too scarce to confirm correlations between such substances as anthocyanins, lignans, resveratrol or quercetin and the concentration of adiponectin [82,83,84,85,86,87,88]. A study by Tucakovic et al. [82] revealed that a four-week diet enhanced with the Queen Garnet plum, which is rich in anthocyanins, increased adiponectin concentrations by an average of 3.83 μg/mL (*p* = 0.048). Yang et al. [83] also demonstrated a correlation between anthocyanin supplementation for 12 weeks and adiponectin concentrations compared to placebo. Comparable results were also obtained by Jeong et al. [84], who demonstrated that the daily consumption of black raspberry for 12 weeks was associated with a considerable increase in adiponectin concentrations.

Resveratrol is another polyphenol influencing adiponectin. A randomized case-control study by Tomé-Carneiro et al. [85] revealed that a six-month dietary supplementation with grape extract increased adiponectin concentrations by 9.6% (*p* = 0.01) compared to placebo. The correlation was confirmed by a systematic review and a meta-analysis of 10 randomized case-control studies which showed that resveratrol supplementation contributed to a marked increase in adiponectin concentrations [89].

A study by Shahi et al. [86] revealed that lignans present in sesame seeds also had strong anti-inflammatory properties, and a diet enriched with sesame seeds significantly increased adiponectin concentrations in T2DM patients.

In addition, quercetin, mainly found in onion skin, also presented a beneficial effect in terms of adiponectin concentrations. Kim et al. [87] conducted a study in a group of women with excessive body weight. Quercetin supplementation for 12 weeks resulted in a significant increase in adiponectin concentration by 3.3 μg/mL compared to baseline. Additionally, a study by Rezvan et al. [88] including women with PCOS showed that oral quercetin supplementation increased the expression of the AdipoR1 and AdipoR2 adiponectin receptors.

#### 2.2.4. Dairy Products

Dairy products also seem to exert positive effects on adiponectin concentrations. However, there is a paucity of studies to confirm such a relationship. Nevertheless, due to the anti-inflammatory properties of such products, especially natural yoghurt, we may presume their positive effect on adiponectin concentrations [90]. A positive correlation between adiponectin concentrations and dairy products may be related to the content of milk fat, whey protein, vitamin D, calcium, potassium, magnesium and the reciprocal relations between those components [91]. A cross-sectional study including 612 Japanese individuals revealed that a diet characterized by the consumption of milk products was associated with higher adiponectin concentrations [91]. Yannakoulia et al. [92] also demonstrated a correlation between adiponectin concentrations and a dietary pattern characterized by a high intake of whole-grain cereal products and low-fat milk products. Similar results were obtained by Niu et al. [93], but a correlation was only observed for low-fat milk products. The correlation with high-fat milk products remained statistically insignificant. Fragopoulou et al. [5] also confirmed that the intake of low-fat milk products was positively correlated with adiponectin concentrations.

#### 2.2.5. Alcohol

Moderate alcohol consumption also proved to be beneficial in relation to adiponectin concentrations. The prospective Nurses’ Health Study [13] revealed that the respective total adiponectin and HMW adiponectin levels were 28% and 45% higher in women from the highest quintile of alcohol intake (0.62–7.19 servings/d) compared to women who consumed no alcohol. Pischon et al. [12] and Bell et al. [94] also showed a significant positive correlation between moderate alcohol consumption and serum adiponectin concentrations. A similar correlation was reported in a study by Beulens et al. [95]. They noted that moderate alcohol intake for four weeks contributed to an increase in total adiponectin concentrations of 12.5% (*p* < 0.001). Furthermore, a cross-sectional study by Nova et al. [96] revealed that the relationship between adiponectin concentrations and moderate alcohol consumption was particularly visible in the case of wine (*p* = 0.017). The possible beneficial effect of this type of alcohol on adiponectin concentrations may be due to the content of polyphenols which are characterized by strong anti-inflammatory properties. However, some studies revealed no effect of alcohol consumption on the concentration of this adipokine [14,97,98]. Additionally, completely different results were observed as regards excessive alcohol consumption which was distinctly associated with low adiponectin concentrations [97,99]. The mechanism through which chronic heavy alcohol consumption is associated with reduced adiponectin concentrations is related to increased oxidative stress, the intensified expression of CYP2E1 (Cytochrome P450 2E1) and the reduced expression of PPARγ [100]. Detailed results of studies on the positive effects of dietary patterns on the concentration of adiponectin are described in Table 1.

To conclude, adherence to the Mediterranean diet is related to particularly significant beneficial effect on serum adiponectin concentrations. The reciprocal relations between the components of this diet (i.e., monounsaturated fatty acids, polyunsaturated omega-3 fatty acids, fiber and polyphenols) and their individual properties contribute to such a positive effect. Moreover, the advantages of the Mediterranean diet are visible regardless of body weight, health status and sex. The properties of the DASH diet also seem promising. However, more research is necessary to provide an explicit confirmation of its positive effect on adiponectin concentrations. Furthermore, the plant-based diet and low-calorie diet seem to be beneficial in the context of adiponectin concentrations. Additionally, moderate alcohol consumption and dairy product intake seem to be of importance in terms of the regulation of adiponectin concentrations. However, more research is needed to determine the influence of such products on the expression of adiponectin.

## 3. Diet-Related Factors with a Negative Influence on Adiponectin Concentrations

### 3.1. Dietary Structure

Incorrect dietary patterns, mainly including the Western diet, are also highly correlated with adiponectin concentrations. The Western diet is mostly characterized by the high content of highly processed food, red meat and refined cereal products [101]. A systematic review and meta-analysis of 12 observational studies revealed an association of the diet with a chronic inflammation [102]. Likewise, a cross-sectional study by Jafari-Vayghan et al. [101] demonstrated that the Western diet was negatively correlated with adiponectin concentrations. Comparable results were also obtained by Alves-Santos et al. [103] in a group of pregnant women. Adherence to the Western dietary pattern was negatively related to intra-gestational adiponectin concentrations.

#### 3.1.1. High-Carbohydrate and Low-Fat Diet

The proportions between individual macronutrients also seem to be significant in relation to adiponectin concentrations. The results were particularly unfavorable in the case of a diet containing high amounts of carbohydrates and low amounts of lipids. A randomized case-control study by Song et al. [44] showed a significant reduction in adiponectin concentrations by 9.4% in a group of persons following a low-fat high-carbohydrate diet compared to those whose diets included the standard content of those macronutrients. Ruth et al. [104] also demonstrated that a high-carbohydrate low-fat diet was associated with a lower mean increase in adiponectin concentrations compared to a high-fat low-carbohydrate diet. Comparable results were obtained by Rajaie et al. [105] who noted that following a high-carbohydrate diet was linked to a significant reduction in the blood content of adiponectin. The authors suggested that the excessive consumption of carbohydrates activated proinflammatory factors by contributing to hyperglycemia and hypertriglyceridemia resulting in reducing adiponectin concentrations. The analysis of correlation in a study by Meshkini et al. [11] revealed that adiponectin concentrations in the circulation were negatively related to the amount of carbohydrates in the diet. A study by Pischon et al. [12] also showed that a 5% increase in energy obtained from carbohydrates instead of lipids was associated with a significant reduction in adiponectin concentrations by 0.59 mg/L. Kasim-Karakas et al. [106] also demonstrated that following a low-fat diet contributed to a reduction in adiponectin concentrations. The change from eucaloric diet providing 35% of energy from lipids into eucaloric diet providing 15% of energy from lipids was associated with a 14% reduction in adiponectin concentrations in healthy postmenopausal women. Similar results were obtained in a study by Murillo-Ortiz et al. [107], who found that women who consumed a diet with a reduced lipid content (12% of energy) for six months were characterized by significantly lower adiponectin concentrations compared to women whose diets included the standard amount of lipids (30% of energy).

#### 3.1.2. High Glycemic Index of the Diet

A high dietary glycemic index also had a negative influence on adiponectin concentrations. The mechanisms of such a correlation have not been precisely described. However, such a dietary pattern may exert a negative effect on adiponectin concentrations by increased glycemia which may contribute to the reduction in the expression of adiponectin in the adipose tissue and activate mTORC1 (mammalian target of rapamycin complex). It is also possible that a high glycemic index reduces adiponectin concentrations by increasing the amount of the adipose tissue [11]. Cerman et al. [108] found a negative correlation between the glycemic index of a diet and serum adiponectin concentrations. A study by Meshkini et al. [11] also revealed that adiponectin concentrations were negatively correlated with the glycemic index and glycemic load of a diet. Furthermore, a high glycemic index was one of stronger negative predictors of the concentration of this adipokine. AlEssa et al. [61] demonstrated that adiponectin concentrations decreased along with increase in the glycemic index. Similar results were obtained by Pischon et al. [12] who observed that each increase of the glycemic index by 1 unit was related to a significant decrease in adiponectin concentration by 1.32 mg/L. The correlations between adiponectin concentrations and dietary glycemic index were also demonstrated in studies by Pereira et al. [62], Qi et al. [63] and Loh et al. [109] in patients with T2DM.

### 3.2. Nutrients and Products Included in the Diet

Saturated fatty acids, trans fatty acids, monosaccharides and disaccharides are the components of the Western diet which are responsible for its proinflammatory properties. Moreover, a high red meat content, particularly processed meat, is a factor which negatively affects adiponectin concentrations. The influence of these dietary components on this adipokine seems to be direct, similarly to those of the remaining dietary components [110].

#### 3.2.1. Saturated Fatty Acids and Trans Fatty Acids

Negative influence on adiponectin concentrations was predominantly observed in case of saturated fatty acids which may affect adiponectin expression in adipocytes via interaction with transcription factors [111]. According to Prates et al. [27], the consumption of large amounts of SFA (Saturated Fatty Acids) was negatively correlated with adiponectin concentrations. A high consumption of total lipids and cholesterol was also negatively interrelated with adiponectin concentrations. A study by Lepsch et al. [110] revealed a correlation between SFA consumption during pregnancy and reduced adiponectin concentrations. Furthermore, Haidari et al. [111] reported that a negative correlation between SFA and adiponectin concentrations was statistically significant both in patients with asthma and in the healthy controls. A negative influence on adiponectin expression was also observed in case of trans fatty acids which were significantly linked to reduced adiponectin as reported in the Nurses’ Health Study [13]. Women from the highest quartile of the consumption of trans fatty acids had significantly lower adiponectin concentrations compared to women from the lowest quartile. A similar correlation was also observed in relation to HMW adiponectin. Additionally, a study by Pereira et al. [62] revealed that a lower consumption of trans fatty acids was associated with significantly higher adiponectin concentrations.

#### 3.2.2. Monosaccharides and Disaccharides

There is a paucity of research on correlations between a diet rich in monosaccharides and disaccharides and adiponectin concentrations. However, as fructose largely contributes to the accumulation of visceral adipose tissue, it may be presumed that individuals whose diets are characterized by high fructose content may present a higher tendency towards reduced adiponectin concentrations [112]. The assumption was confirmed by a study conducted by Rezvani et al. [112]. They reported that participants who consumed large amounts of fructose were characterized by significantly reduced adiponectin concentrations. Moreover, a similar correlation was also observed in relation to glucose. A negative influence of these monosaccharides on adiponectin concentrations was observed only after 10 weeks of the intervention. Therefore, it may be assumed that only the long-term use of a diet including high monosaccharide content exerts a negative effect on this adipokine. Similar results were obtained by Pollock et al. [113], who studied a group of 559 adolescents. They reported that a diet rich in fructose was associated with significantly lower adiponectin concentrations. Besides, Magalhaes et al. [114] demonstrated that adiponectin was also influenced by the consumption of sucrose which is a disaccharide. Participants whose diets were rich in sucrose were characterized by significantly reduced adiponectin concentrations (<0.35 μg/mL). The correlation occurred both in individuals with nonalcoholic fatty liver disease and in healthy participants. Furthermore, hypoadiponectinemia was associated with the consumption of sweets and sweetened beverages by healthy individuals.

#### 3.2.3. Red Meat

Similar to monosaccharides and disaccharides, a paucity of research has been performed to investigate correlations between red meat consumption and adiponectin concentrations. However, because of the proinflammatory properties of red meat, particularly processed meat, it may be assumed that its high dietary content adversely affects adiponectin concentrations [115]. A study by Fargnoli et al. [13] revealed that adiponectin concentrations decreased with an increasing red meat to poultry ratio in the diet. Ley et al. [116] also demonstrated the presence of a correlation between the consumption of red meat and adiponectin concentrations. Women from the highest quartile of the total consumption of unprocessed and processed red meat had significantly lower adiponectin concentrations compared to women from the lowest quartile. Interestingly, Chai et al. [115] found that the consumption of processed red meat was significantly related to reduced adiponectin concentrations in women. Surprisingly, such a correlation was not observed in men. Additionally, the authors noted that BMI might be an intermediate factor between red meat consumption and adiponectin concentrations. A diet rich in red meat may contribute to body weight increase and promote adipose tissue deposition, which may induce obesity-related inflammation. Detailed results of studies on the negative effects of dietary patterns on the concentration of adiponectin are described in Table 2.

To conclude, a negative influence on adiponectin concentrations seems to be exerted mainly by a high glycemic index diet and by the Western diet, characterized by the consumption of red meat, particularly processed meat, and products which provide high amounts of saturated fatty acids, trans fatty acids, fructose and sucrose. All components of this diet seem to have a direct negative effect on adiponectin concentrations. However, more research is necessary to confirm whether the high dietary content of red meat, monosaccharides and disaccharides is directly linked to the expression of adiponectin. Moreover, proportions between proteins, lipids and carbohydrates in the diet are of enormous importance, as high-carbohydrate and low-fat diets are significantly related to hypoadiponectinemia.

## 4. Conclusions

**Figure 1:**
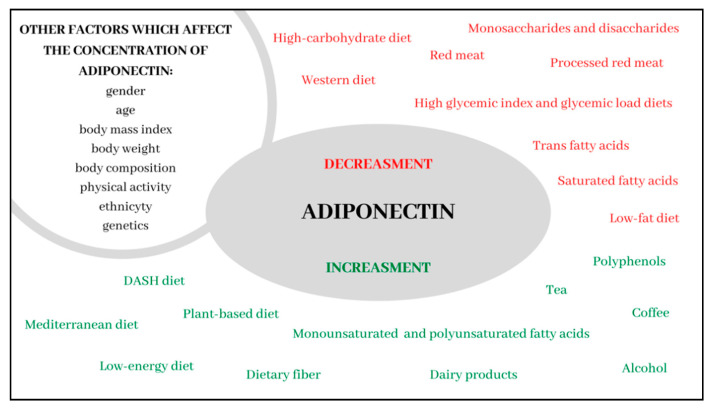
Summary of the influence of dietary factors on the concentration of adiponectin.

Dietary factors play an extremely important role in the regulation of adiponectin concentrations. Adherence to the Mediterranean dietary pattern is one of the strongest modulators of its concentration. The presence of monounsaturated fatty acids, polyunsaturated omega-3 fatty acids, fiber and polyphenols make the Mediterranean diet particularly beneficial. Moreover, it seems likely that the relationship between MD and the prevention of civilization diseases, such as cancer, cardiovascular disease and metabolic disorders, may result from its influence on the concentration of this adipokine. It seems that the DASH diet, diet based on plant products and diet with reduced energy value also contain dietary patterns responsible for the increase in adiponectin concentrations. Additionally, the moderate consumption of alcohol and milk products appear to be significant in terms of exerting a beneficial influence on the regulation of its concentrations. Conversely, high glycemic index and glycemic load, a high consumption of red meat, particularly processed meat, and products rich in saturated fatty acids, trans fatty acids, and fructose and sucrose are factors which adversely affect adiponectin concentrations (the summary of the influence of dietary factors on the concentration of adiponectin constitutes Figure 1). Therefore, it seems that hypoadiponectinemia is particularly associated with dietary patterns typical of the Western diet and high-carbohydrate low-fat diet. Due to the paucity of data to confirm the correlation between individual dietary components, it is necessary to conduct more research to determine which dietary components are directly related to the expression of adiponectin.

## Figures and Tables

**Table 1:** Dietary patterns and the concentration of adiponectin (AD)—A positive effect.

Author/Reference	Year	Study Design	Sample	Results
**HEALTHY DIET**				
Fargnoli et al. [13]	2008	Prospective cohort study	1922 women free of CVD, diabetes and cancer, aged 30–55 y	Total AD concentration was 24% higher (15.68 ± 1.03 μg/mL vs. 12.61 ± 1.03 μg/mL; *p* < 0.0001) and HMW AD was 32% higher (5.71 ± 1.04 μg/mL vs. 4.34 ± 1.04 μg/mL; *p* < 0.0001) in women from the highest quartile of adherence to AHEI compared to women from the lowest quartile.
Volp et al. [14]	2016	Cross-sectional study	157 apparently healthy men and women, aged 18–35 y	A correlation between the Healthy Eating Index and AD concentrations (*r* = 0.20074; *p* = 0.02).
**THE MEDITERRANEAN DIET**				
Mantzoros et al. [17]	2006	Cross-sectional study	987 diabetic women, aged 30–55 y	Higher adherence to the MD was associated with markedly higher AD concentrations compared to the lowest adherence (6.91 ± 1.06 μg/mL vs. 5.49 ± 1.04 μg/mL; *p* < 0.01).
Fragopoulou et al. [5]	2010	Cross-sectional study	532 men and women free of CVD, aged > 18 y	Higher adherence to the MD was associated with markedly higher AD concentrations compared to the lowest adherence (4.8 ± 2.0 μg/mL vs. 3.4 ± 1.9 μg/mL; *p* < 0.001). A correlation between scores obtained for the MD and AD concentrations (women: rho = 0.156; *p* = 0.02), (men: rho = 0.130; *p* = 0.02).
Schwingshackl et al. [21]	2014	A systematic review and meta-analysis of 17 interventional studies	2300 men and women, aged 25–77 y	Adherence to the rules of the MD was related to significantly higher AD concentrations compared to the control diet (WMD: 1.69 mg/mL, 95% CI 0.27, 3.11; *p* = 0.02).
Maiorino et al. [15]	2016	Randomized control study	215 men and women with newly diagnosed T2DM, aged > 18 y	Following the MD for a year was associated with an increase in total AD concentrations by 43% (6.12 vs. 8.80 μg/mL; *p* < 0.001) and HMV AD by 54% (2.41 vs. 3.72 μg/mL; *p* < 0.01).
Sureda et al. [16]	2018	Cross-sectional study	598 men and women, aged 12–65 y	Adherence to the rules of the MD was related to significantly higher AD concentrations compared to non-adherence 13.1 ± 6.7 μg/mL vs. 9.5 ± 2.4 μg/mL; *p* < 0.05). No correlation found in women and adolescents of both sexes.
Spadafranca et al. [20]	2018	Cohort study	99 normal weight, pregnant women, aged 25–43 y	Women from the highest tertile of adhering to the MD were characterized by a lower decrease in the percentage of AD concentrations compared to women from the lowest tertile (10% ± 11% vs. −34% ± 3%; *p* = 0.01).
Luisi et al. [19]	2019	Interventional study	36 men and women, aged > 18 y	Following the MD enhanced with 40 g/d of extra virgin olive oil was associated with increased AD concentrations (increase by 0.6 ± 0.26 μg/mL; *p* < 0.01 in a group with normal body weight and an increase by 1.6 ± 0.2 μg/mL; *p* < 0.01 in a group with excessive body weight).
Kouvari et al. [18]	2020	Prospective cohort study	3042 apparently healthy men and women, aged > 18 y	Higher adherence to the MD was associated with markedly higher AD concentrations compared to the lowest adherence 4.8 ± 2.0 μg/mL vs. 3.4 ± 1.9 μg/mL; *p* < 0.001)
**THE DASH DIET**				
Nilsson et al. [25]	2019	Cross-sectional study	112 women, aged 65–70 y	The highest tertile of adherence to the DASH diet was associated with markedly higher AD concentrations compared to the lowest tertile (12.9 ± 3.3 μg/mL vs. 11.5 ± 3.4 μg/mL; *p* = 0.008).
**PLANT-BASED DIET**				
Kahleova et al. [30]	2011	Randomized control study	74 men and women with T2DM, aged 30–70 y	An increase in total AD by 19% (95% CI 7.5–25.4; *p* < 0.05) and HMV AD by 15% (95% CI 3.6–23.6; *p* < 0.05) after 24 weeks of following a vegetarian diet.
Ambroszkiewicz et al. [29]	2018	Cross-sectional study	117 prepubertal children, aged 5–10 y	Following a vegetarian diet was associated with a significantly higher adiponectin to leptin ratio (0.70 (0.37–0.93) vs. 0.39 (0.28–0.74); *p* = 0.005) compared to the traditional diet.
Mirmiran et al. [65]	2019	Randomized cross-over clinical trial	31 men and women with T2DM, aged 50–75 y	The consumption of two servings of pulses instead of red meat for eight weeks was associated with an increase in AD concentrations (10.5 ± 3.0 μg/mL vs. 13.1 ± 3.0 μg/mL; *p* < 0.05).
**Lovrenčić et al.** [31]	2020	Case-control study	76 non-obese men and women, aged 19–59	Following a vegetarian diet was associated with significantly higher AD concentrations compared to the traditional diet (*p* = 0.03). No correlation in men.
**LOW-CALORIE DIET**				
Christiansen et al. [41]	2010	Randomized controlled trial	79 obese men and women, aged 18–45 y	VLCD diet (800 kcal/d) was associated with a 19% increase in AD concentrations after 12 weeks (*p* < 0.01).
Abbenhardt et al. [42]	2013	Randomized controlled trial	439 overweight or obese postmenopausal women, aged 50–75 y	AD concentrations increased by 9.5% after 12 months of following LCD (12.4 μg/mL (11.3–13.5) vs. 13.5 μg/mL (12.5–14.6); *p* < 0.0001) and by 6.6% (12.8 μg/mL (11.7–13.9) vs. 13.6 μg/mL (12.5–14.8); *p* = 0.0001) as a result of combining LCD with physical activity.
Bouchonville et al. [40]	2014	Randomized controlled trial	107 obese men and women, aged ≥65 y	Calorie reduction of the diet by 500–700 kcal contributed to an increase in AD concentration by 8.9 μg/mL (3.5–14.8); *p* < 0.01), while the combination of reduction diet and physical activity contributed to an AD increase by 6.5 μg/mL (0.8–12.3; *p* = 0.02).
Salehi-Abargouei et al. [43]	2015	Systematic review and meta-analysis of interventional trials (13 interventional studies)	937 men and women, aged 20–75 y	The use of LCD was associated with an increase in AD concentration (Hedges’ g = 0.34, 95 % CI 0.17–0.50; *p* < 0.001), especially if the diet was followed for at least 16 weeks (Hedges’ g for ≤ 16 weeks = 0.48, 95% CI: 0.12–0.83; *p* = 0.01, (Hedges’ g for > 6 weeks = 0.30, 95 % CI: 0.11–0.48; *p* = 0.002).
**Monda et al.** [39]	2020	Interventional study	20 obese men and women, aged 20–60 y	The use of ketogenic VLCD for 8 weeks was associated with a significant increase in AD concentrations both in women (12.44 ± 1.07 μg/mL vs. 27.3 ± 1.33 μg/mL; *p* < 0.05), and in men (9.23 ± 0.7 μg/mL vs. 32.67 ± 1.6 μg/mL; *p* < 0.05).
**POLYUNSATURATED FATTY ACIDS**				
Fargnoli et al. [13]	2008	Prospective cohort study	1922 women, free of CVD, diabetes and cancer, aged 30–55 y	Women from the group characterized by the lowest ratio of PUFA to SFA consumption had significantly higher total AD (12.66 ± 1.03 μg/mL vs. 11.47 ± 1.03 μg/mL; *p* = 0.01) and HMW (4.19 ± 1.04 μg/mL vs. 3.60 ± 1.03 μg/mL; *p* = 0.005) compared to women with the highest ratio.
Kalgaonkar et al. [58]	2011	Randomized, prospective study	36 women with PCOS, aged 20–45 y	The consumption of walnuts and almonds significantly increased AD concentrations (walnuts: 9.5 ± 1.6 μg/mL vs. 11.3 ± 1.8 μg/mL; *p* = 0.0241; almonds: 10.1 ± 1.5 μg/mL vs. 12.2 ± 1.4 μg/mL; *p* = 0.0262).
Nadjarzadeh et al. [53]	2015	Randomized double-blind placebo-controlled clinical trial.	84 women with polycystic ovary syndrome, aged > 18 y	Omega-3 supplementation (180 mg EPA and 120 mg DHA) for eight weeks significantly increased AD concentrations (4.44 ± 1.92 μg/mL vs. 5.62 ± 2.68 μg/mL; *p* < 0.005).
Gomes et al. [56]	2015	Randomized double-blind, placebo-controlled trail	20 men and women with T2DM, aged 30–65 y	Supplementation with 3 g of ALA increased AD concentrations after 60 days (10.61 ± 6.53 μg/mL vs. 15.01 ± 11.68 μg/mL; *p* = 0.01).
Balfegó et al. [51]	2016	Pilot randomized trial	35 men and women with T2DM, aged 40–70 y	Introducing 10 g of sardines into the diet (five times a week for six months) was associated with a significant increase in AD concentrations (2.1 ± 0.3 μg/mL vs. 3.0 ± 0.3 μg/mL; *p* = 0.04)
Barbosa et al. [48]	2017	Randomized, double-blind placebo-controlled clinical trial	80 men and women with at least one cardiovascular risk factor, aged 30–74 y	Omega-3 supplementation (3 g/d) for two months significantly increased AD concentrations (14.8 ± 10.0 μg/mL vs. 18.2 ± 12.1 μg/mL; *p* = 0.021).
Mazaherioun et al. [49]	2017	Randomized, placebo-controlled, double-blind clinical trial	88 men and women with T2DM, aged 30–65 y	Omega-3 supplementation (2.7 g/d) significantly increased AD concentrations (5.09 ± 2.79 μg/mL vs. 5.58 ± 3.13 μg/mL; *p* < 0.001).
Mejia-Montilla et al. [54]	2018	Prospective study	195 women with PCOS, aged > 18 y	N-3 supplementation (180 mg EPA and 120 mg DHA) significantly increased AD concentrations (3.9 ± 1.1 μg/mL vs. 5.3 ± 1.4 μg/mL; *p* = 0.001), both in women with HOMA-IR <3.5 (3.9 ± 1.1 μg/mL vs. 5.3 ± 1.4 μg/mL; *p* < 0.0001), and in those with HOMA-IR >3.5 (4.1 ± 1.1 μg/mL vs. 5.6 ± 1.3 μg/mL; *p* = 0.005).
Song et al. [52]	2018	Double-blind randomized controlled trial	201 healthy men and women, aged > 40 y	An increase in AD concentrations over 12 weeks as a result of omega-3 supplementation at a dose of: 3.1 g/d (5.79 ± 2.68 μg/mL vs. 6.36 ± 2.64 μg/mL; *p* < 0.05), 6.2 g/d (5.72 ± 2.07 μg/mL vs. 6.87 ± 2.58 μg/mL; *p* < 0.01) and 12.4 g/d (5.81 ± 2.13 μg/mL vs. 7.43 ± 2.63 μg/mL; *p* < 0.01).
Bahreini et al. [47]	2018	A systematic review and meta-analysis of interventional trials (10 randomized controlled trails)	177 men and women with T2DM, aged > 18 y	An increase in AD concentrations by 0.57 μg/mL as a result of omega-3 supplementation (95% CI 0.15–1.31; *p* = 0.01).
Becic et al. [45]	2018	A systematic review and meta-analysis of interventional trials (10 randomized controlled trails)	460 men and women with prediabetes and T2DM, aged > 18 y	An increase in AD concentrations by 0.48 μg/mL as a result of omega-3 supplementation (95% Cl 0.27–0.68; *p* < 0.00001).
Haidari et al. [55]	2020	Randomized open-labeled controlled clinical trial	41 women with PCOS, aged 18–45 y	An increase in AD concentrations over 12 weeks as a result of supplementation with 30 g of ground linseed (13.04 ± 3.36 μg/mL vs. 17.36 ± 4.1 μg/mL; *p* = 0.002).
Khorrami et al. [50]	2020	Randomized double-blind, placebo-controlled study	80 overweight or obese men and women with atrial fibrillation, aged > 50 y	An increase in AD concentrations over eight weeks as a result of supplementation with 2 g/d of fish oil (11.88 ± 6.94 μg/mL vs. 13.15 ± 7.33 μg/mL; *p* = 0.026).
Yang et al. [59]	2020	A systematic review and meta-analysis of randomized clinical trials (3 randomized controlled trails)	823 men and women, aged > 18 y	The consumption of walnuts significantly increased AD concentrations (WMD: 0.440 μg/mL; 95% CI: 0.323 to 0.557; *p* < 0.001).
**DIETARY FIBER**				
Qi et al. [63]	2005	Cross-sectional study	780 men with T2DM, aged 40–75 y	Men from the highest quartile of dietary fiber consumption had significantly higher AD concentrations compared to men from the lowest quartile (17.3 μg/mL vs. 14.2 μg/mL; *p* = 0.006).
Mantzoros et al. [17]	2006	Cross-sectional study	987 diabetic women, aged 30–55 y	The consumption of whole-grain cereal products was associated with significantly higher AD concentrations (6.11 ± 1.06 μg/mL vs. 4.92 ± 1.05 μg/mL; *p* < 0.01).
Fargnoli et al. [13]	2008	Prospective cohort study	1922 women free of CVD, diabetes and cancer, aged 30–55 y	Women from the highest quartile of cereal fiber consumption were characterized by significantly higher total AD concentrations (14.73 ± 1.03 μg/mL vs. 13.36 ± 1.04 μg/mL; *p* < 0.04) and AD HMW (5.32 ± 1.04 μg/mL vs. 4.56 ± 1.04 μg/mL; *p* < 0.02) compared to women from the lowest quartile.
Pereira et al. [62]	2016	Observational, cross-sectional study	43 men and women, 18–60 y	A higher consumption of fiber included in vegetables and fruit was associated with higher AD concentrations (r = 0.50; *p* = 0.0007). The concentrations of adiponectin were 4.7 μg/mL (*p* = 0.03) higher in individuals from the highest quartile of cereal fiber consumption compared to participants from the lowest quartile.
AlEssa et al. [64]	2016	Cross-sectional study	2458 women, free of diabetes, aged 43–70 y	Women from the highest quintile of total fiber (*p* < 0.001), cereal fiber (*p* < 0.001), fruit fiber (*p* = 0.014) and vegetable fiber (*p* = 0.011) consumption had significantly higher AD concentrations compared to women from the lowest quintile.
**CURCUMIN**				
Campos-Cervantes et al. [77]	2011	Randomized, single blind, placebo-controlled trial	50 obese men, aged 25–30 y	An increase in AD concentrations after six and 12 weeks of supplementation with 500 mg of curcumin (after six weeks: 16.0 μg/mL vs. 18.5 μg/mL; *p* < 0.01 and after 12 weeks: 16.0 μg/mL vs. 18. μg/mL; *p* < 0.02).
Panahi et al. [76]	2016	Randomized controlled trial	117 men and women, aged > 18 y	An increase in AD concentrations after eight weeks of supplementation with 1000 mg of curcumin (12.67 ± 2.13 μg/mL vs. 21.28 ± 4.40 μg/mL; *p* < 0.001).
Mirhafez et al. [78]	2019	Randomized, double blind, placebo-controlled, cross-over trial	65 men and women with nonalcoholic fatty liver disease, aged > 18 y	Supplementation with 250 mg/d of curcumin for wight weeks caused a significant increase in AD concentrations (14.35 ± 7.72 μg/mL vs. 18.23 ± 9.75 μg/mL; *p* < 0.001).
Adibian et al. [79]	2019	Randomized, double blind, placebo-controlled trial	44 men and women with T2DM, aged 40–70 y	Supplementation with 1500 mg/d of curcumin for 10 weeks caused a significant increase in AD concentrations (52.0 ± 8.0 μg/mL vs. 64.0 ± 3.0 μg/mL; *p* < 0.0001).
Clark et al. [81]	2019	A systematic review and meta-analysis of interventional trials (10 randomized controlled trails)	652 men and women with type 2 diabetes, prediabetes subjects, obese men or with metabolic syndrome, aged 18–84 y	Supplementation with curcumin caused a significant increase in AD concentrations compared to placebo (WMD: 0.82 Hedges’ g; 95% CI 0.33–1.30; *p*˂0.001). A particularly beneficial effect of at least 10 weeks of supplementation (WMD: 1.05 Hedges’ g; 95% CI: 0.64 to 1.45; *p* ˂ 0.001).
Akbari et al. [80]	2019	Systematic review and meta-analysis of randomized controlled trials (21 randomized controlled trails)	1646 men and women with metabolic syndrome	An increase in AD concentrations after supplementation with curcumin (SMD 1.05; 95% CI 0.23–1.87; *p* = 0.01).
**ANTHOCYANINS**				
Jeong et al. [84]	2014	Prospective randomized double-blind study	77 men and women with metabolic syndrome, aged 18–75 y	Daily black raspberry consumption for 12 weeks was associated with an increase in AD concentrations (5.7 ± 5.1 μg/mL vs. 7.7 ± 5.0 μg/mL; *p* < 0.05).
Tucakovic et al. [82]	2018	Randomized, double-blind, placebo-controlled, cross-over trial	20 apparently healthy men and women, aged 18–65 y	Supplementation with the Queen Garnet plum for four weeks increased AD concentrations by the average of 3.83 μg/mL (*p* = 0.048).
Yang et al. [83]	2020	Randomized controlled trial	160 men and women with T2DM or prediabetes	Anthocyanin supplementation for 12 weeks was associated with an increase in AD concentrations compared to placebo (increase by 0.46 μg/mL; *p* = 0.038).
**RESVERATROL**				
Tomé-Carneiro et al. [85]	2013	Triple-blind, placebo-controlled clinical trial	75 men and women, aged > 18 y	Supplementation with grape extract for six months increased AD concentrations by 9.6% (*p* = 0.01).
Mohammadi-Sartang et al. [89]	2017	Systematic review and meta-analysis of randomized controlled trials (9 randomized controlled trails)	590 men and women, aged > 18 y	Resveratrol supplementation significantly increased AD concentrations (WMD: 1.10 μg/mL, 95% CI 0.88, 1.33; *p* < 0.001)
**QUERCETIN**				
Kim et al. [87]	2016	Randomized double-blind, placebo-controlled study	37 healthy overweight and obese women	AD increase after 12 weeks of quercetin supplementation (3.6 ± 2.0 μg/mL vs. 6.9 ± 2.3 μg/mL; *p* < 0.05).
Rezvan et al. [88]	2018	Randomized double-blind, placebo-controlled study	81 women with PCOS, aged 20–40 y	An increased expression of the AD receptors (AdipoR1 and AdipoR2) after 12 weeks of supplementation with 1 g/d of quercetin (*p* < 0.01).
**LIGNANS**				
Shahi et al. [86]	2017	Randomized double-blind, placebo-controlled study	48 men and women with T2DM, aged 30–60 y	AD increase after eight weeks of supplementation with 200 mg/d of sesamin (6.21 ± 1.33 μg/mL vs. 7.34 ± 2.88 μg/mL; *p* = 0.024).
**COFFEE**				
Williams et al. [70]	2008	Prospective cohort study	982 women with T2DM and 1058 nondiabetic women	The consumption of ≥4 cups of coffee daily was associated with significantly higher AD compared to the consumption of <1 cup a week (women with T2DM: 7.7 vs. 6.1 μg/mL; *p* = 0.002, nondiabetic women: 15.0 vs. 13.2 μg/mL; *p* = 0.04).
Kempf et al. [68]	2010	Single-blind clinical trial	47 men and women, free of T2DM, aged 18–65 y	The consumption of eight cups of coffee daily was associated with significantly higher AD concentrations compared to consuming no coffee (8421 (6634–11256) ng/mL vs. 7957 (6317, 10901) ng/mL; *p* < 0.05).
Imatoh et al. [71]	2011	Cross-sectional study	665 men, aged > 18 y	The consumption of ≥3 cups of coffee daily was associated with significantly higher AD compared to consuming no coffee (6.9 ± 3.3 μg/mL vs. 6.0 ± 2.6 μg/mL; *p* < 0.01).
Yamashita et al. [69]	2012	Cross-sectional study	3317 men and women, aged 35–69 y	The consumption of ≥4 cups of coffee daily was associated with significantly higher AD compared to the consumption of <1 cup a week (7.23 (6.84–7.65) μg/mL vs. 6.58 (6.40–6.76) μg/mL; *p* = 0.005).
**GREEN TEA**				
Hsu et al. [73]	2008	Randomized, double-blind, placebo-controlled clinical trial	78 obese women, aged 16–60 y	An increase in AD concentrations after 12 weeks of supplementation with 400 mg of green tea extract (18.9 ± 6.7 μg/mL vs. 21.4 ± 8.7 μg/mL; *p* < 0.01).
Fragopoulou et al. [5]	2010	Cross-sectional study	532 men and women free of CVD, aged > 18 y	A correlation was found between green tea consumption and AD concentrations (rho = 0.108; *p* = 0.04).
Liu et al. [74]	2014	Randomized, double-blind, and placebo-controlled trial	102 men and women with T2DM, aged 20–65 y	An increase in AD concentrations after 16 weeks of supplementation with 500 mg of green tea extract (20.2 ± 5.1 μg/mL vs. 21.7 ± 5.1 μg/mL; *p* < 0.046).
Chen et al. [72]	2016	Randomized, double-blind trial	92 obese women, aged 20–60 y	An increase in AD concentrations after 12 weeks of supplementation with 856.8 mg of green tea extract (20.9 ± 11.0 μg/mL vs. 24.0 ± 10.7 μg/mL; *p* = 0.009).
**DAIRY PRODUCTS**				
Yannakoulia et al. [92]	2008	Cross-sectional study	196 apparently healthy women, aged 18–84 y	A correlation occurred between AD and a dietary pattern rich in low-fat dairy and whole-grain cereal products (*r* = 0.15; *p* = 0.04).
Niu et al. [93]	2013	Cross-sectional one-year longitudinal study	938 apparently healthy men and women, aged > 18 y	The consumption of low-fat milk products (58.9–375 g/d) was associated with significantly higher AD concentrations compared to no consumption of such products (8.3 (7.8, 8.9) μg/mL vs. 7.3 (6.9, 7.6) μg/mL; *p* < 0.01).
Fragopoulou et al. [5]	2010	Cross-sectional study	532 man and women free of CVD, aged > 18 y	A correlation occurred between the consumption of low-fat milk products and AD concentrations (rho = 0.119, *p* = 0.04).
Bahari et al. [91]	2018	Cross-sectional study	612 men and women, 35–69 y	A diet characterized by the higher consumption of milk products was associated with higher AD concentrations (4.78 (3.24, 7.38) μg/mL vs. 3.68 (2.42, 6.12) μg/mL; *p* = 0.004).
**ALCOHOL**				
Pischon et al. [12]	2005	Prospective cohort study	532 men, aged 40–75 y	Men from the highest quintile of AD concentrations (>24.9 μg/mL) consumed significantly more alcohol (16.2 ± 1.06 g/d vs. 13.05 ± 0.7 g/d) compared to men from the lowest quintile of AD concentrations (<10.6 μg/mL); *p* = 0.006). A correlation occurred between AD concentrations and alcohol consumption (*r* = 0.14; *p* = 0.002).
Fargnoli et al. [13]	2008	Prospective cohort study	1922 women free of CVD, diabetes and cancer, aged 30–55 y	Total AD concentrations were 28% higher (16.01 ± 1.03 vs. 12.50 ± 1.03; *p* < 0.0001) and HMW AD concentrations were 45% higher (6.10 ± 1.04 vs. 4.21 ± 1.03; *p* < 0.0001) in women from the highest quintile of alcohol consumption compared to those who consumed no alcohol.
Beulens et al. [95]	2007	Randomized, controlled, cross-over trial	17 apparently healthy men, aged 18–40 y	Moderate alcohol consumption (32 g/d) for four weeks caused an increase in total AD concentrations by 12.5% (*p* < 0.001).
Bell et al. [94]	2015	Prospective cohort study	2855 men and women, aged 40–63 y	Alcohol consumption was cross-sectionally associated with AD concentrations (β = 0.003; *p* < 0.001).
Nova et al. [96]	2019	Observational cross-sectional study	240 men and women, aged 55–85 y	Wine consumption was associated with higher AD (*β* = 204, 95% CI: 37–370; *p* = 0.017).

Abbreviations: CVD, Cardiovascular disease; y, years; AHEI, Alternate Healthy Eating Index; AD, adiponectin; AD HMW, high-molecular-weight adiponectin; MD, Mediterranean diet; WMD, Weighted Mean Difference, T2DM, Type 2 Diabetes Mellitus; DASH, Dietary Approach to Stop Hypertension; VLCD, very low calorie diet; LCD, low calorie diet; PUFA, Polyunsaturated Fatty Acids; SFA, Saturated Fatty Acids; PCOS, polycystic ovary syndrome; EPA, eicosapentaenoic acid; DHA, docosahexaenoic acid; ALA, α-linolenic acid; HOMA-IR, Homeostatic Model Assessment of Insulin Resistance.

**Table 2:** Dietary patterns and the concentration of adiponectin (AD)—A negative effect.

Author/Reference	Year	Study Design	Sample	Results
**THE WESTERN DIET**				
Jafari-Vayghan et al. [101]	2015	Cross-sectional study	150 apparently healthy men and women, aged 25–50 y	Adherence to the Western dietary pattern was negatively correlated with AD concentrations (*r* = −0.19; *p* = 0.02).
Alves-Santos et al. [103]	2018	Prospective cohort study	173 pregnant women free of infectious and chronic diseases, aged 20–40 y	Adherence to the Western dietary pattern was negatively correlated with AD concentrations during pregnancy (high vs. low tertile of adherence: *β* = −1.11; 95% CI −2.00, −0.22; *p* < 0.05).
**HIGH-CARBOHYDRATE LOW-FAT DIET**				
Pischon et al. [12]	2005	Prospective cohort study	532 men, aged 40–75 y	A 5% increase in energy obtained from carbohydrates instead of lipids was associated with reduction in AD concentrations by 0.59 μg/mL (*p* = 0.05).
Kasim-Karakas et al. [106]	2006	Interventional study	22 healthy postmenopausal women, aged > 50 y	Following the eucaloric LFHC diet was linked to a reduction in AD concentrations (16.3 ± 2.1 μg/mL to 14.2 ± 2.0 μg/mL; *p* < 0.05).
Rajaie et al. [105]	2013	Randomized cross-over clinical trial	30 overweight or obese women with metabol ic syndrome, aged 20–65 y	Following HCD for 6 weeks was linked to AD concentration reduction by 1.68 ± 2.30 μg/mL (10.6 ± 0.3 μg/mL vs. 8.9 ± 0.3 μg/mL; *p* < 0.001).
Ruth et al. [104]	2013	Randomized clinical trial	55 obese men and women, aged 21–62 y	Following an HFLC diet for 12 weeks was related to a significant increase in AD concentrations (+0.40 ± 0.66 μg/mL, *p* = 0.045).
Song et al. [43]	2016	Randomized controlled interventional study	93 women and men aged 21–76 years	AD decreased by 9.4% (*p* = 0.008) in individuals following an LFHC diet compared to those following a diet with a moderate fat content.
Murillo-Ortiz et al. [107]	2017	Randomized controlled clinical trial	100 postmenopausal women with breast cancer, aged >48 y	Following a diet with the reduced fat content (12% of energy) for 6 months was associated with reduced AD concentrations (21.23 ± 14.32 μg/mL vs. 16.05 ± 10.25 μg/mL; *p* < 0.001).
Meshkini et al. [11]	2018	Cross-sectional study	89 apparently healthy men and women, aged 18–75 y	AD concentrations were negatively correlated with the amount of carbohydrates in the diet (*r* = −0.24, *p* = 0.02).
**GLYCEMIC INDEX AND GLYCEMIC LOAD OF THE DIET**				
Qi et al. [63]	2005	Cross-sectional study	780 men with T2DM, aged 40–75 y	AD concentrations significantly lower in the highest quintile of the GI of the diet compared to the lowest GI (14.3 μg/mL vs. 16.4 μg/mL; *p* = 0.005). AD concentrations significantly lower in the highest quintile of the GL of the diet compared to the lowest GL (14.1 μg/mL vs. 17.2 μg/mL; *p* = 0.004).
Pischon et al. [12]	2005	Prospective cohort study	532 men, aged 40–75 y	Men from the highest quintile of AD concentrations (>24.9 μg/mL) were characterized by a significantly higher GL of the diet (124.7 ± 2.1 vs. 128.5 ± 1.0; *p* = 0.04) compared to men from the lowest quintile of AD concentrations (<10.6 μg/mL); *p* = 0.006). Each GL increment by 1 unit was associated with AD reduction by 1.32 μg/mL (*p* = 0.02).
Loh et al. [109]	2013	Cross-sectional study	305 T2DM men and women, aged 19–75 y	A negative correlation between the GI of the diet and AD concentrations (*β* = −0.272, 95% CI −0.262–0.094; *p* < 0.001).
Cerman at tal. [108]	2016	Cross-sectional study	86 men and women apparently healthy or with acne vulgaris, aged > 18 y	A negative correlation between the GI of the diet and AD concentrations (*r* = −0.212; *p* = 0.049).
Pereira et al. [62]	2016	Observational, cross-sectional study	43 men and women, aged 18–60 y	A high GI of the diet was negatively correlated with AD concentrations (*r* = −0.47; *p* = 0.0017).
AlEssa et al. [64]	2016	Cross-sectional study	2458 women, free of diabetes, aged 43–70 y	Women from the highest quintile of diet GI had significantly lower AD concentrations compared to women from the lowest quintile (11.7 (11.2, 12.3) μg/mL vs. 12.9 (12.4, 13.4) μg/mL; *p* < 0.001).
Meshkini et al. [11]	2018	Cross-sectional study	89 apparently healthy women and men, aged 18–75 y	AD concentrations were negatively correlated with diet GI (*r* = −0.43; *p* < 0.001) and GL (*r* = −0.29; *p* = 0.007). A high GI diet was one of stronger negative predictors of AD concentrations (*β* = −0.176, *p* = 0.04).
**SATURATED FATTY ACIDS AND TRANS FATTY ACIDS**				
Fargnoli et al. [13]	2008	Prospective cohort study	1922 women free of CVD, diabetes and cancer, aged 30–55 y	Women from the highest quartile of trans fatty acid consumption had significantly lower total AD concentrations (13.5 ± 1.03 μg/mL vs. 14.96 ± 1.03 μg/mL, *p* = 0.0002) and AD HMW concentrations (4.49 ± 1.04 μg/mL vs. 5.20 ± 1.04 μg/mL; *p* = 0.0008) compared to women from the lowest quartile.
Haidari et al. [111]	2014	Case-control study	94 men and women apparently healthy or with asthma	AD concentrations were negatively correlated with SFA consumption in persons with asthma (*r* = −0.319; *p* = 0.033) and in healthy individuals (*r* = −0.356; *p* = 0.016).
Pereira et al. [62]	2016	Observational, cross-sectional study	43 men and women, aged 18–60 y	AD concentrations were negatively related to the consumption of trans fatty acids (*r* = −0.4, *p* = 0.008).
Prates et al. [27]	2016	Cross-sectional study	122 men and women with T1DM, aged > 18 y	AD concentrations were negatively correlated with SFA consumption (*r* = −0.25, *p* = 0.004), total fat consumption (*r* = −0.20, *p* = 0.02), and cholesterol consumption (*r* = −0.20, *p* = 0.021).
Lepsch et al. [110]	2016	Prospective cohort study	201 pregnant women, aged 22–31 y	A negative correlation between SFA consumption and AD concentrations (*β* = −41.039; *p* = 0.008).
**MONOSACCHARIDES AND DISACCHARIDES**				
Pollock et al. [113]	2012	Cross-sectional study	559 adolescents, aged 14–18 y	A diet with high fructose content was associated with significantly lower AD concentrations (8.4 ± 0.4 μg/mL vs. 9.1 ± 0.4 μg/mL; *p* = 0.033).
Rezvani et al. [112]	2013	Double-blind parallel arm study	32 overweight or obese men and women, aged 40–72 years	Participants who consumed high quantities of glucose (*p* = 0.028) and fructose (*p* = 0.0011) had significantly decreased AD concentrations after 10 weeks.
Magalhaes et al. [114]	2014	Cross-sectional study	60 obese women with nonalcoholic fatty liver disease or apparently healthy, aged >20 y	Diet rich in sucrose was significantly related to low AD concentrations (<0.35 μg/mL) in healthy women (*p* = 0.054) and in women with NAFLD *(p* = 0.045). Diet rich in sweets (*p* = 0.046) and sweetened beverages (*p* = 0.054) was significantly correlated with low AD concentrations in healthy women (<0.35 μg/mL).
**RED MEAT**				
Fargnoli et al. [13]	2008	Prospective cohort study	1922 women free of CVD, diabetes and cancer, aged 30–55 y	Women from the highest quartile of the red meat to poultry consumption ratio had significantly lower total AD concentrations (13.24 ± 1.03 μg/mL vs. 14.52 ± 1.03 μg/mL, *p* = 0.02) compared to women from the lowest quartile.
Ley et al. [116]	2014	Prospective cohort study	21700 women, aged 30–55 y	Women from the highest quartile of the consumption of red meat (13.7 (13.1, 14.3) μg/mL vs. 15.0 (14.4, 15.6) μg/mL, *p* = 0.003), unprocessed red meat (14.0 (13.4, 14.5) μg/mL vs. 15.0 (14.4, 15.6) μg/mL; *p* = 0.01) and processed red meat (13.9 (13.3, 14.5) μg/mL vs. 15.0 (14.4, 15.6) μg/mL; *p* = 0.007) had significantly lower AD concentrations compared to women from the lowest quartile.
Chai et al. [115]	2017	Case-control study	1223 men and women free of cancer, aged 45–75 y	The consumption of red processed meat was associated with reduced AD concentrations in women (*β* = −0.082; *p* = 0.005).

Abbreviations: AD, adiponectin; AD HMW, high-molecular-weight adiponectin; y, years; LFHC, low-fat high-carbohydrate diet, HFLC, high-fat low-carbohydrate diet, HCD, high calorie diet, IG, glycemic index, GL, glycemic load, SFA, Saturated Fatty Acids; CVD, cardiovascular disease, NAFLD, nonalcoholic fatty liver disease.
